# Kernelized partial least squares for feature reduction and classification of gene microarray data

**DOI:** 10.1186/1752-0509-5-S3-S13

**Published:** 2011-12-23

**Authors:** Walker H Land, Xingye Qiao, Daniel E Margolis, William S Ford, Christopher T Paquette, Joseph F Perez-Rogers, Jeffrey A Borgia, Jack Y Yang, Youping Deng

**Affiliations:** 1 Department of Bioengineering, Binghamton University, Binghamton, NY 13902, USA; 2 Department of Mathematical Sciences, Binghamton University, Binghamton, NY 13902, USA; 3 Department of Biochemistry, Rush University Medical Center, Chicago, IL 60612, USA; 4 Department of Radiation Oncology Massachusetts General Hospital and Harvard Medical School Boston, MA 02114, USA; 5 Department of Internal Medicine, Rush University Cancer Center, Rush University Medical Center, Chicago, IL 60612, USA

## Abstract

**Background:**

The primary objectives of this paper are: 1.) to apply Statistical Learning Theory (SLT), specifically Partial Least Squares (PLS) and Kernelized PLS (K-PLS), to the universal "feature-rich/case-poor" (also known as "large *p *small *n*", or "high-dimension, low-sample size") microarray problem by eliminating those features (or probes) that do not contribute to the "best" chromosome bio-markers for lung cancer, and 2.) quantitatively measure and verify (by an independent means) the efficacy of this PLS process. A secondary objective is to integrate these significant improvements in diagnostic and prognostic biomedical applications into the clinical research arena. That is, to devise a framework for converting SLT results into direct, useful clinical information for patient care or pharmaceutical research. We, therefore, propose and preliminarily evaluate, a process whereby PLS, K-PLS, and Support Vector Machines (SVM) may be integrated with the accepted and well understood traditional biostatistical "gold standard", Cox Proportional Hazard model and Kaplan-Meier survival analysis methods. Specifically, this new combination will be illustrated with both PLS and Kaplan-Meier followed by PLS and Cox Hazard Ratios (CHR) and can be easily extended for both the K-PLS and SVM paradigms. Finally, these previously described processes are contained in the Fine Feature Selection (FFS) component of our overall feature reduction/evaluation process, which consists of the following components: 1.) coarse feature reduction, 2.) fine feature selection and 3.) classification (as described in this paper) and prediction.

**Results:**

Our results for PLS and K-PLS showed that these techniques, as part of our overall feature reduction process, performed well on noisy microarray data. The best performance was a good 0.794 Area Under a Receiver Operating Characteristic (ROC) Curve (AUC) for classification of recurrence prior to or after 36 months and a strong 0.869 AUC for classification of recurrence prior to or after 60 months. Kaplan-Meier curves for the classification groups were clearly separated, with *p*-values below 4.5e-12 for both 36 and 60 months. CHRs were also good, with ratios of 2.846341 (36 months) and 3.996732 (60 months).

**Conclusions:**

SLT techniques such as PLS and K-PLS can effectively address difficult problems with analyzing biomedical data such as microarrays. The combinations with established biostatistical techniques demonstrated in this paper allow these methods to move from academic research and into clinical practice.

## Introduction

One of the most popular and challenging topics in bioinformatics research is gene selection from microarray data because it involves both statistical processing as well as biological interpretation. The statistical problems are daunting because of the large number of represented genes relative to the small number of samples. This provides a prime opportunity to over-fit the data during the model building process. Biology is a significant component because identifying significant genes representative of a given clinical endpoint is a critical step toward understanding the biological process. Several consequences arise as a result of the statistical over-fitting problem. Very large Receiver Operating Characteristic (ROC) Area Under the Curve (AUC) values can be achieved on both training and validation data sets, but the results provided by these trained Complex Adaptive Systems (CAS) frequently fail to generalize to data sets other than training and validation sets. Furthermore, these CAS system designs do not necessarily operate on similar data sets with larger representative samples. Different CAS solutions may produce different gene sets from the same set of microarray data. Consequently, any CAS should first attempt to achieve some sort of generalization ability. Secondly, because of the over-fitting problem described above, each proposed feature (or gene) reduction CAS generally is based on a unique theoretical analysis, which means that how these separate CAS are connected is not well understood. Consequently, this difficulty results in the same problem stated above: different algorithms will generate different prognostic gene sets using the same microarray data. This means that developing an underlying theory for feature selection would help to understand these algorithms as well as classify which of these are the "most" useful for gene selection. Song [1] presents a BAHSIC algorithm which claims to address this unifying algorithm principle proposal. BAHSIC defines a class of backward (BA) elimination feature selection algorithms that uses kernels and the Hilbert-Schmidt Independence Criterion (HSIC) [2]. Song demonstrates that the BAHSIC algorithm encompasses the following well-known feature selection algorithms: (1) Pearson's correlation coefficient [3,4], (2) *t*-test [5], (3) signal-to-noise ratio [6], (4) Centroid [7,8], (5) Shrunken Certroid [9,10], and finally, (6) ridge regression [11]. These collective results suggest that the Evolutionary Programming driven Support Vector Machine (EP-SVM) [12,13] with a choice of similarity, sum and product kernels might be a good wrapper/classification candidate for gene selection. This paper adapts a method, summarized in the methods section, originally developed for the social sciences and subsequently adapted to chemometrics, called Partial Least Squares (PLS) to this "feature-rich/case-poor" environment, as subsequently described, by theoretically attempting to eliminate those features which do not contribute to the "best" chromosome marker for lung cancer.

### Background of lung cancer

Lung cancer is the leading cause of death in cancer patients worldwide. The American Cancer Society predicts that 156,940 people will fall victim to the disease in 2011, accounting for 27% of all cancer deaths [14]. The 5-year survival rate of lung cancer patients is 16% primarily due to late stage diagnosis. Of the 221,130 estimated cases that will be diagnosed in 2011, 85% will have late stage tumors (stages II, III, IV) that have begun to advance. For these patients, treatment often includes surgical resection of tumors where possible, post-operative radiation and adjuvant chemotherapy. The 5-year survival rate for early stage (stage I), non-small cell lung cancer (NSCLC) patients is 53% [14] and treatment often only includes surgical resection [15]. However, 35%-50% of these patients will suffer a relapse of the disease within 5 years of surgery [16]. Post-operative chemotherapy can, in most cases, improve survival in early stage cancer patients. But its use is controversial. Doctors currently lack a validated and clinically accepted method to predict which patients are at a high risk of recurring cancer [17]. Those patients that are at a high risk of recurrence might benefit from post-operative adjuvant chemotherapy, whereas those patients that are at a low risk can be spared the side effects of chemotherapy [18].

### Data set description and modifications

The experiments designed used the gene expression profiles of 442 lung adenocarcinomas compiled by Shedded *et al. *[19]. These samples were compiled from six institutions and originally handled by a consortium that included: the University of Michigan (177 samples), the H. Lee Moffit Cancer Center (79 samples), the Dana-Farber Cancer Institute (82 samples) and the Memorial Sloan-Kettering Cancer Center (104 samples).

It is important to note that in Dobbin *et al. *[20] these samples were shown to be comparable because the variability in gene expression values can be attributed more to the biology of the samples than to the institution effect. As a result, the data can be combined for the purposes of our analysis despite being processed at different institutions. Furthermore, none of the patients in the study received pre-operative chemotherapy or radiation and at least two years of follow up information was available. Tumor samples were required to contain a minimum of 60% tumor cellularity for inclusion in the study with most containing 70%-90%.

Gene expression profiles of all samples were quantified using the Affymetrix Human Genome-U133A GeneChip. The resulting CEL files generated at each of the four institutions were quantile normalized using the NCI_U133A_61L array as a reference. Final expression values were calculated using the DChip software (Build version February 2006) using the default settings. Each sample is characterized by 22,283 probes/genes (also referred to as features in this paper) as well as a host of clinical covariates including age, gender, and T/N cancer stage. A few minor discrepancies were found in the probe data obtained from the caArray website. First, probe 207140 at contained expression values of "NA" for all patients in the study. To mitigate this problem, the data corresponding to this probe were removed prior to our analysis. Secondly, patients Moff 18351, Moff 2362A and Moff 3009D did not have expression values for the 222086_s_at probe. In lieu of removing this probe entirely, the data for these patients were assigned an expression value equal to the mean (18.37114) of that probe's expression values across all other patients. The CEL files, DChip normalized expression values and clinical information for all patients involved in this study are available through the caArray website https://array.nci.nih.gov/caarray/project/details.action?project.id=182. Other work with this data set is described in [18] and [19].

### Experimental design for 3 and 5 Years

To address the clinical issue of determining risk of recurrence delineated above, two classification experiments were designed. The first experiment classified NSCLC patients as "high risk" if cancer were likely to recur within 3 years of surgery and "low risk" otherwise. The 3 year cut-off was chosen because the majority of patients that do relapse will do so within the first 3 years [16]. The second experiment objective was to classify patients as "high risk" if cancer were likely to recur within 5 years of surgery, and "low risk" otherwise. This cut-off was chosen because, in current clinical practice, a patient that does not recur cancer within 5 years is often considered "cancer free" due to the low chance of recurrence after that time [21].

The first experiment contained 295 patients obtained from the Shedden *et al. *[19] data set. This subset contained all patients for which cancer recurred as well as those that survived beyond 3 years (without recurrence). For purposes of training and validating the algorithms discussed in this paper, patients for which cancer recurred within 3 years (of surgery) are considered recurrent (high risk) and those that had recurrent cancer, or survived without recurrence beyond 3 years, are considered non-recurrent (low risk).

The second experiment was composed of 257 patients, which were also obtained from the Shedden *et al. *[19] data set. All recurrent patients, as well as those that survived without recurrence beyond 5 years, were included in this analysis. Patients for which cancer recurred within 5 years of surgery were considered recurrent (high risk). Those that recurred, or survived without recurrence beyond 5 years, were considered non-recurrent (low risk). Finally, it also should be noted that Shedden *et al. *[19] showed that the use of the clinical covariates age, gender and tumor stage during analysis improved the performance of most classifiers. Consequently, it was required that all patients included in the two experiments described above have available clinical information pertaining to the features: age, gender and tumor stage.

## Methods

### Overview of Feature Reduction/Classification Process

**Figure 1:**
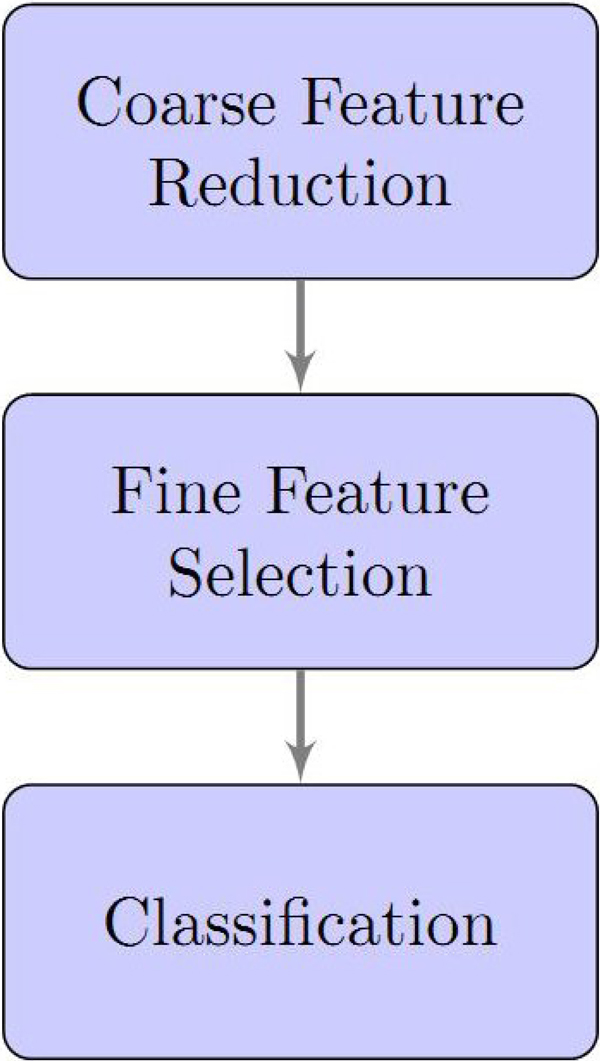
**Feature (probe) reduction process**. Description of feature reduction process

Microarray data sets have a significant feature-rich/case-poor problem which can lead to over-fitting (i.e. models that produce excellent results on the training data exist, but none of which may be valid and have good performance on the test data) unless the number of features are significantly reduced prior to the generation of any classification or prediction model. The objective of this three-step process is to identify those significant features which are most useful in producing an accurate classification or prediction model. The process of feature reduction/classification is depicted in Figure 1, and consists of a Coarse Feature Reduction (CFR) process, followed by a Fine Feature Selection (FFS) process and then classification.

#### Coarse Feature Reduction

The automated CFR employees a simple two sample *t*-test followed by variance pruning (cut-off based on coefficient of variation). It is a simple process to remove lot of probes that are not useful for classification, *i.e*., those not considered statistically significant to classification. See [22-24] for details on variance pruning.

#### Partial Least Squares

This section contains a brief, heuristic overview of Partial Least Squares (PLS). PLS is an extension of least squares regression (LSR). In LSR, the response variable *y *is predicted from *p *coordinates and *n *observations, denoted by *X *= {*x*_1_,*x*_2_, ...*x*_*n*_}^*T*^, where each $x_{i}\in {ℜ}^{p}$. PLS finds "new variables" through the construction of specific combinations of the original coordinates. These "latent variables" explain both the *y *response as well as the covariate space and are denoted by the following expressions:

(1) $$X=t_{1}p_{1}+t_{2}p_{2}+...+t_{s}p_{s}+\varepsilon$$

(2) $$y=t_{1}q_{1}+t_{2}q_{2}+...+t_{s}q_{s}+\zeta$$

where:

• *t*_*s *_= the *s*^*th *^latent variable (or conjugate vector; a *n *by 1 column vector). Generally most of the variability is characterized by *M *latent variables with a maximum of *M *= 5 required for most problems.

• *p*_*s *_and *q*_*s *_= the *s*^*th *^weight vectors (*p*_s _is a 1 by *p *row vector, *q*_*s *_is scalar).

• *ε*, ζ = small errors in the remaining parts not explained by the latent variables.

For this microarray data set, we began with 271 features after CFR and reduced this set to a minimum of 1 latent variable and a maximum of 5 latent variables (see Results section). Therefore, the principle advantage of PLS for a problem of this type is its ability to handle a very large number of features: a fundamental problem of a feature-rich/case-poor data set. PLS then performs a least-squares fit (LSF) onto these latent variables, where this LSF is a linear combination that is highly correlated with the desired *y *response while, at the same time, accounting for the feature space variability. A summary of the features and advantages of PLS follows:

• PLS algorithms are very resistant to over-fitting, when compared to LSR, and are fast and reasonably easy to implement.

• For most problems with few data points and high dimensionality where PLS excels, a least squares solution may not be possible due to the singularity problem.

• PLS regression maps the original data into a lower-dimensional space using a *W *projection matrix and computes a least squares solution in this space. See the algorithm below for the definition of *W*.

• What makes PLS especially interesting for biomedical and data mining applications is its extension using kernels, which leads to kernelized PLS (K-PLS), similar to the treatment in SVM.

• PLS may be considered a better principal component analysis (PCA).

- The first key difference from PCA is that PLS computes an orthogonal factorization of the input vector *X and *response *y *(note: *y *can also be a vector) in the process of computing the projection matrix *W*.

- The second key difference from PCA is that the least squares model for K-PLS is based on approximation of the input and response data, not the original data.

- PLS and PCA use different mathematical models to compute the final regression coefficients. Specifically, the difference between PCA and PLS is that a new set of basis vectors (similar to the eigenvectors of *X*^*T*^*X *in PCA) is not a set of succession of orthogonal directions that explain the largest variance in data, but rather are a set of conjugate gradient vectors in the correlation matrices which span a Krylov space.

An algorithm of PLS paradigm follows:

1. Let: *X*_1 _= *X,y*_1 _= *y*

2. For *m *= *1 *to *M*, where *M *= the desired number of latent variables, do:

(a) Compute direction of maximum variance         *w*_*m *_= (*X*_*m*_)^*T*^*y*_*m*_

(b) Project *X *onto *w*         *t*_*m *_= *X*_*m *_*w *_*m*_

(c) Normalize *t*         *t*_m _= *t*_*m*_/|*t*_*m*_|

(d) Deflate *X*         *X*_*m*+1 _= *X*_*m*_-*t*_m_(*t*_*m*_)^*T*^*X*_*m*_

(e) Deflate *y*         *y*_*m*+1 _= *y*_*m*_-*t*_m_(*t*_*m*_)^*T*^*y*_*m*_

(f) Normalize *Y *after deflation         *y*_*m*+1 _= *y*_*m*+1_/|*y*_*m*+1_|

3. Finally, compute the regression coefficients using latent variables:         *β *= *W*(*T*^*T*^*XW*)^-1^*T*^*T*^*y*

where:

• *w*_*m *_is the *m*^*th *^column vector of *W*

• *t*_*m *_is the *m*^*th *^column vector of *T*

• *X*_*m *_and *y*_*m *_are the input matrix and response vector that are being deflated, and *β *is the linear regression coefficient vector. A geometric representation of part of the algorithm and the insight of deflation can be seen in Figure 2.

**Figure 2:**
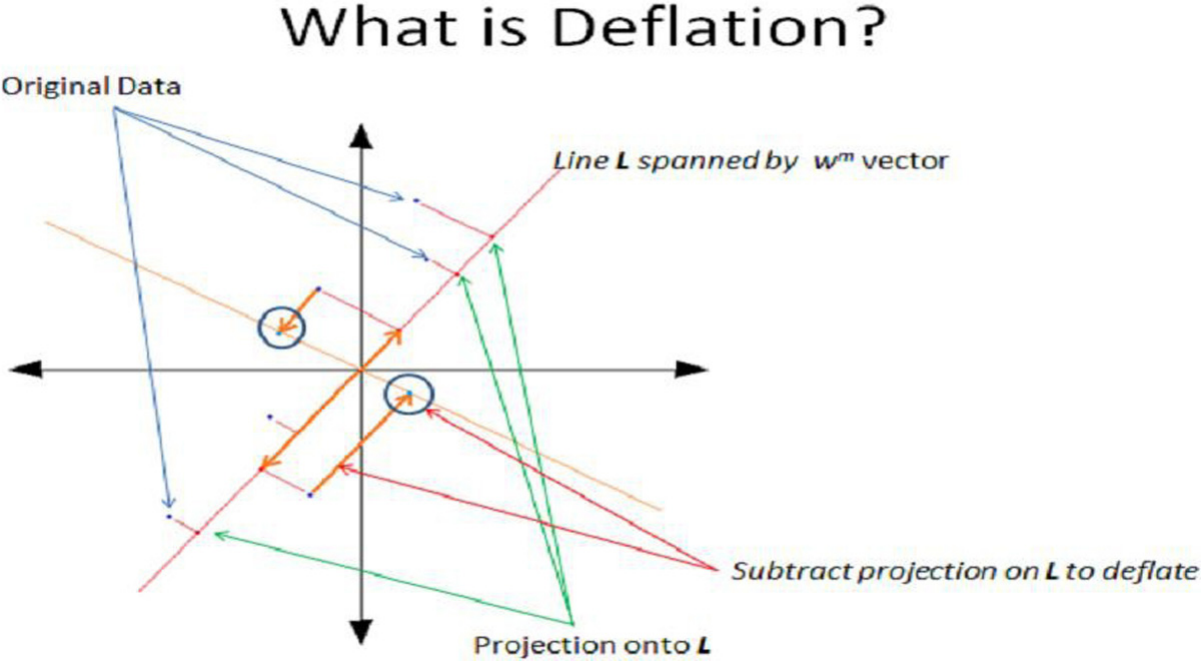
**Deflation**. The geometric interpretation of the 'deflation' step in the PLS Algorithm. This 'Deflation' effectively removes one dimension by projecting the data onto a subspace that is one dimension less than the row number of the current data matrix, and orthogonal to the vector *W*^*m*^.

#### Kernelized Partial Least Squares

Non-linear relationships between variables may be found by embedding this data into a kernel induced feature space. See [25] for a good reference of kernel learning. This kernel "trick" is used in PLS and is called K-PLS. Consider now a mapping *ϕ*, which maps any data vector from the sample space to some other (possibly infinite dimensional) Euclidean space H (feature space):

(3) $$\phi :{ℜ}^{n}\to H$$

The mapping will "recode" the data set as:

(4) $$\left\{\left(\phi \left(x_{1}\right),y_{1}\right),\left(\phi \left(x_{2}\right),y_{2}\right),\cdots \left(\phi \left(x_{n}\right),y_{n}\right)\right\}$$

This mapping of the data set is from non-linear input space to a linear feature space. That is, although the environment data representation in the input *X *space is non-linear, after the data are processed by the *ϕ *mapping, the data characterized by this mapping is linear in H, with the happy result that linear techniques may be used on the mapped data while preserving the non-linear properties represented in the input space. This mapping is accomplished, as previously stated, by using a valid kernel function.

Adding this kernel-induced capability to the PLS approach means that a real time, non-linear optimal training method now exists which can be used to perform computer aided diagnosis. A second advantage of this approach is that a kernel function *K*(*x*_1_,*x*_2_) computes the inner products 〈*ϕ*(*x*_1_),*ϕ*(*x*_2_)〉 in the feature space H directly from the samples *x*_1 _and *x*_2_, without having to explicitly perform the mapping, making the technique computationally efficient. This is especially useful for algorithms that only depend on the inner product of the sample vectors, such as SVM and PLS.

Computationally, kernel mappings have the following important properties: (1) they enable access to exceptionally high (even infinitely) dimensional and, consequently, very flexible feature space, with a correspondingly low time and space computational cost, (2) they solve the convex optimization problem without becoming "trapped" in local minimal and, more importantly, (3) the approach decouples the design of the algorithms from the specifications of the feature space. Therefore, both learning algorithms and specific kernel designs are not as difficult to analyze.

The algorithm used to develop the K-PLS model, is given below. Details can be found in [26].

1. Let *K*_0 _= (*K*_*ij *_= 〈*ϕ*(*x*_*i*_), *ϕ*(*x*_*j*_)〉 = *K*(*x*_*i*_,*x*_*j*_))_(__*ij *= 1,...*n*) _be the *n *by *n *Gram matrix induced by *K*, the selected kernel function corresponding to *ϕ*(·). Let *K*_1 _be the centered form of *K*_0_,*y *be the be the response variable, normalized to have a mean of 0 and a standard deviation of 1, and *M *be the desired number of latent variables.

2. For *m *= *1 *to *M*, do:

(a) *t*_*m *_= *K*_*m *_· *y*_*m*_

(b) $t_{m}=\frac{t_{m}}{\left||t_{m}|\right|}$

(c) $K_{m+1}=\left(I-t_{m}t_{m}^{T}\right)K_{m}\left(I-t_{m}t_{m}^{T}\right)$

(d) $y_{m+1}=y_{m}-\left(t_{m}t_{m}^{T}\right)y_{m}$

(e) $y_{m+1}=\frac{y_{m+1}}{\left||y_{m+1}|\right|}$

3. Finally, compute the regression coefficients using: $a=Y{\left(T^{T}K_{1}^{T}Y\right)}^{-1}T^{T}Y$ where:

• *I *is an *m *× *m *identity matrix

• *K*_*m *_is the Gram matrix

• *t*_*m *_and *y*_*m *_are the *m*^*th *^columns of *T *and *Y *respectively

4. The regression equation then becomes:

(5) $$f\left(x\right)=\overset{n}{\underset{i=1}{\sum }}K_{1}\left(x_{i},x\right)\cdot a_{i}$$

Note *x *is any sample from the testing data to be predicted and *K*_1_(*x*_*i*_,*x*) is element from the centered form of the training/testing kernel matrix.

#### Evolutionary Programming derived K-PLS machines

The particular K-PLS kernel types and kernel parameters were derived using an evolutionary process based on the work of Fogel [27] called Evolutionary Programming (EP). EP is a stochastic process in which a population of candidate solutions is evolved to match the complexity and structure of the problem space.

**Figure 3:**
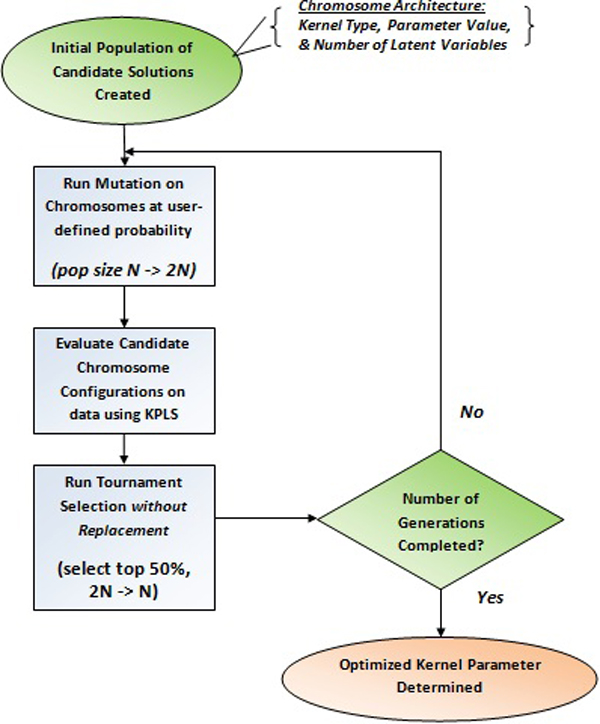
**Evolutionary Programming**. Shown is the process of the Evolutionary Programming optimization technique utilized to find the optimal kernel parameters. The process creates an initial population of candidate solutions (chromosomes) which undergo a stochastic search for the optimal parameter through the sub-processes of mutation and tournament selection of the 'most-fit' genes.

This process iteratively generates, valuates, and selects candidates to produce a near-optimal solution without using gradient information, and is therefore well suited to the task of simultaneously generating both the K-PLS model architecture (kernel) and parameters. Figure 3 and found in more detaA description of this process is shown in Figuil below.

1. *Initial K-PLS parameter population created: *A population of candidate solutions (K-PLS kernel architectures and parameters) is randomly generated.

2. *Mutation of K-PLS machines: *Each of these candidate solutions then is copied and mutated, yielding a solution pool of twice the original size, using the equation given below:

(6) $$v_{i}^{\prime }=v_{i}e^{\left(\frac{1}{\sqrt{2m}}N\left(0,1\right)+\frac{1}{\sqrt{2\sqrt{m}}}N_{i}\left(0,1\right)\right)}$$

where *m *is the total number of configurable parameters being evolved, *N*(0,1) is a standard normal random variable sampled once for all *m *parameters of the *v *vector, and *N*_*i*_(0,1) is a standard normal random variable sampled for *each *of the *m *parameters in the *v *vector.

The second step of this mutation process comprises the updating of each configurable parameter for all elements of the evolving population. If we let the vector γ_*i *_denote these elements for each of the individual member of the population, this update process will be accomplished as follows:

(7) $$\gamma_{i}^{\prime }=y_{i}+Cv_{i}^{\prime }$$

Here *C *is a standard Cauchy random variable. It is used because it has longer tails and offers better mutation performance.

3. *Selection of K-PLS machines: *All elements of this pool are scored using an objective function. These objective function scores are then used to order the candidate solutions from the "most fit" to "least fit." Better results usually are obtained from using tournament selection methodologies. With tournament selection, each candidate solution competes against a random subset of the remaining solutions. Finally, the upper 50% of the solution pool is selected to continue as the basis for the next generation and the remaining 50% are "killed off" (discarded) to reduce the pool to the original population size. This process is generally repeated for a specified number of generations, unless some other "stopping" criteria is used.

For more details on the EP process, refer to our previous work [28].

#### Support Vector Machine and its capacity to reach the global optimum

The K-PLS results were validated by using another kernel-based Statistical Learning Theory model called a Kernelized Support Vector Machine (K-SVM). SVMs was developed by Vapnik [29-31]. Tutorials to SVM can be found in [32] and [25].

The discussion below provides the theoretical explanation for why SVMs can always be trained to a global minimum, and thereby should provide better diagnostic accuracy, when compared with neural network performance trained by back propagation.

Assume there exist *n *experimentally derived observations. Each observation (or training example) consists of a vector *x*_*i *_containing the input pattern and a corresponding known classification *y*_*i*_. The objective of the learning machine is to formulate a mapping *x*_*i *_→ *y*_*i*_. Now consider a set of functions *f*(*x*,*α*) with adjustable parameters *α*, that defines a set of possible mappings *x *→ *f*(*x*,*α*). Here, *x *is given and *α *is chosen. In the case of a traditional neural network of fixed architecture, the *α *values would correspond to the weights and biases. The quantity *R*(*α*), known as the expected risk, associated with learning machines is defined as:

(8) $$R\left(\alpha \right)=\int \frac{1}{2}|y-f\left(x,\alpha \right)|p\left(x,y\right)dxdy$$

where, *p*(*x*, *y*) is an unknown probability density function from which the examples were drawn. This risk function is the expected value of the test (or validation) error for a trained learning machine. It may be shown that the best possible generalization ability of a learning machine is achieved by minimizing *R*(*α*), the expected risk. There exists a error bound of generalization, for binary classification, which holds with the probability of at least 1 - *η*, 0 ≤ *η *≤ 1 for all approximating functions that minimize the expected risk.

(9) $$R\left(\alpha \right)\leq R_{emp}\left(\alpha \right)+\sqrt{\left(\frac{h\left(log\left(\frac{2n}{h}\right)+1\right)-log\left(\frac{\eta }{4}\right)}{n}\right)}$$

The first term on the right hand side, *R*_*emp*_(*α*), is known as the "empirical risk", expressed by:

(10) $$R_{emp}\left(\alpha \right)=\frac{1}{2n}\overset{n}{\underset{i=1}{\sum }}|y_{i}-f\left(x_{i},\alpha \right)|$$

Empirical risk is a measure of the error rate for the training set for a fixed, finite number of observations. This value is fixed for a particular choice of *α *and a given training set {(*x*_*i*_,*y*_*i*_),*i *= 1,2, ···*n*}. The second term in (9) is the "Vapnik-Chervonenkis (VC) confidence interval." This term is a function of the number of training samples *n*, the probability value *η *and the VC dimension *h*. The VC dimension is the maximum number of training samples that can be learned by a learning machine without error for all possible labeling of the classification functions *f*(*x*,*α*), and is, therefore, a measure of the capacity of the learning machine. In traditional neural network implementations, the confidence interval is fixed by choosing a network architecture *a priori*. Neural network training by back-propagation minimizes the empirical risk only.

In contrast to neural network, in a SVM design and implementation, not only is the empirical risk minimized, the VC confidence interval is also minimized by using the principles of structural risk minimization (SRM). Therefore, SVM implementations simultaneously minimize the empirical risk as well as the risk associated with the VC confidence interval, as defined in the above expression. The bound in (9) also shows that as *n *→ ∞, the empirical risk approaches the true risk because the VC confidence interval risk approaches zero. The reader may recall that obtaining larger and larger sets of valid training data would sometimes produce (with a great deal of training experience) a better performing neural network using classical training methods. This restriction is not incumbent on the SRM principle and is the fundamental difference between training neural networks and training SVMs. Finally, because SVMs minimize the expected risk, they provide a global minimum.

#### Measures of similarity for classification provided by various kernels

Understanding what similarity as applied to K-PLS and K-SVM often provides additional insight in proper kernel selection. Therefore, we now consider kernel functions and their application to K-PLS and K-SVMs. K-PLS and K-SVM solutions in non-linear, non-separable learning environments utilize kernel based learning methods. Consequently, it is important to understand the practical implications of using these kernels. Kernel based learning methods are those methods which use a kernel as a non-linear similarity to perform comparisons. That is, these kernel mappings are used to construct a decision surface that is non-linear in the input space, but has a linear image in the feature space. To be a valid mapping, these inner product kernels must be symmetric and also satisfy Mercer's theorem [33]. The concepts described here are not limited to K-PLS and K-SVMs, and the general principles also apply to other kernel based classifiers as well.

A kernel function should yield a higher output from input vectors which are very similar than from input vectors which are less similar. An ideal kernel would provide an exact mapping from the input space to a feature space which was a precise, separable model of the two input classes; however, such a model is usually unobtainable, particularly for complex, real-world problems, and those problems in which the input vector provided contains only a subset of the information content needed to make the classes completely separable. As such, a number of statistically-based kernel functions have been developed, each providing a mapping into a generic feature space that provides a reasonable approximation to the true feature space for a wide variety of problem domains. The kernel function that best represents the true similarity between the input vectors will yield the best results, and kernel functions that poorly discriminate between similar and dissimilar input vectors will yield poor results. As such, intelligent kernel selection requires at least a basic understanding of the source data and the ways different kernels will interpret that data.

Some of the more popular kernel functions are the (linear) dot product (11), the polynomial kernel (12), the Gaussian Radial Basis Function (GRBF) (13), and the Exponential Radial Basis Function (ERBF) (14), which will be discussed below.

**Figure 4:**
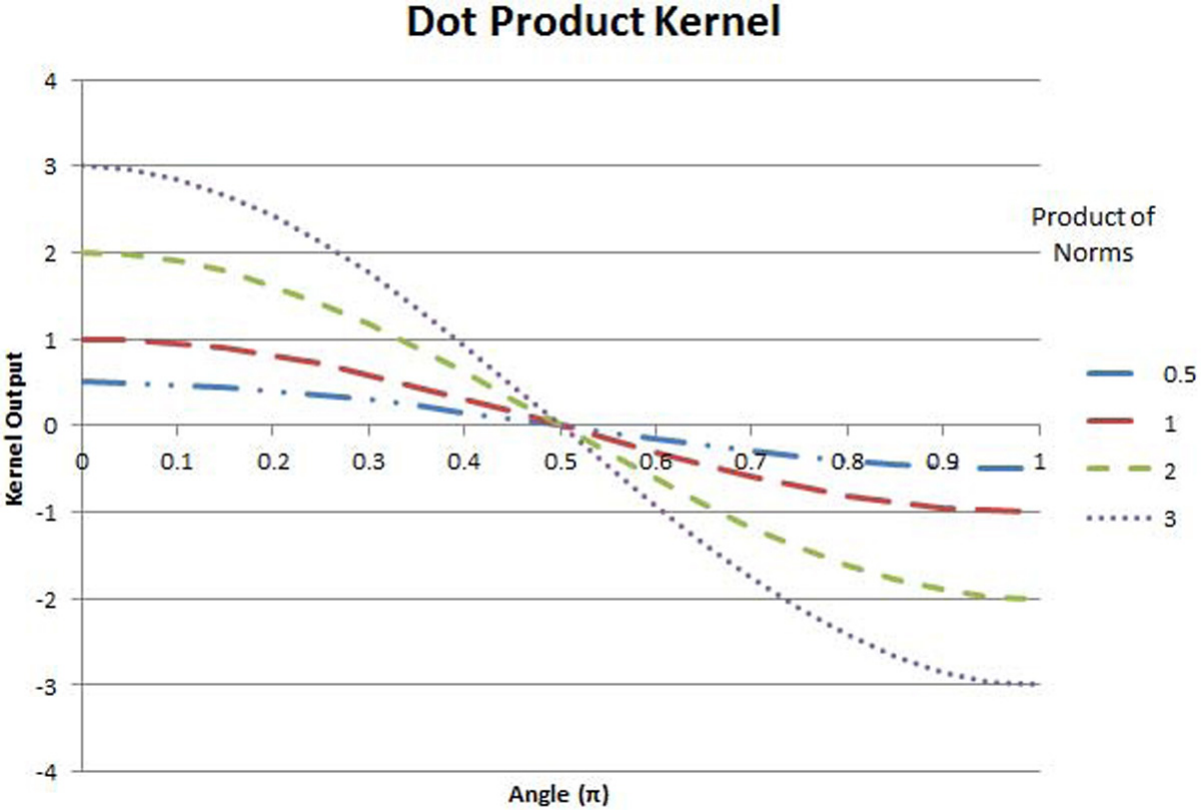
**Dot product kernel**. The outputs of dot product kernel as functions of the angles between vectors. Four functions are depicted in solid-blue, long-dashed-red, short-dashed-green and dotted-purple curves, corresponding to the cases where the product of the norms of the $\vec{u}$ and $\vec{v}$ vectors is equal to 0.5, 1, 2 and 3 respectively.

**Figure 5:**
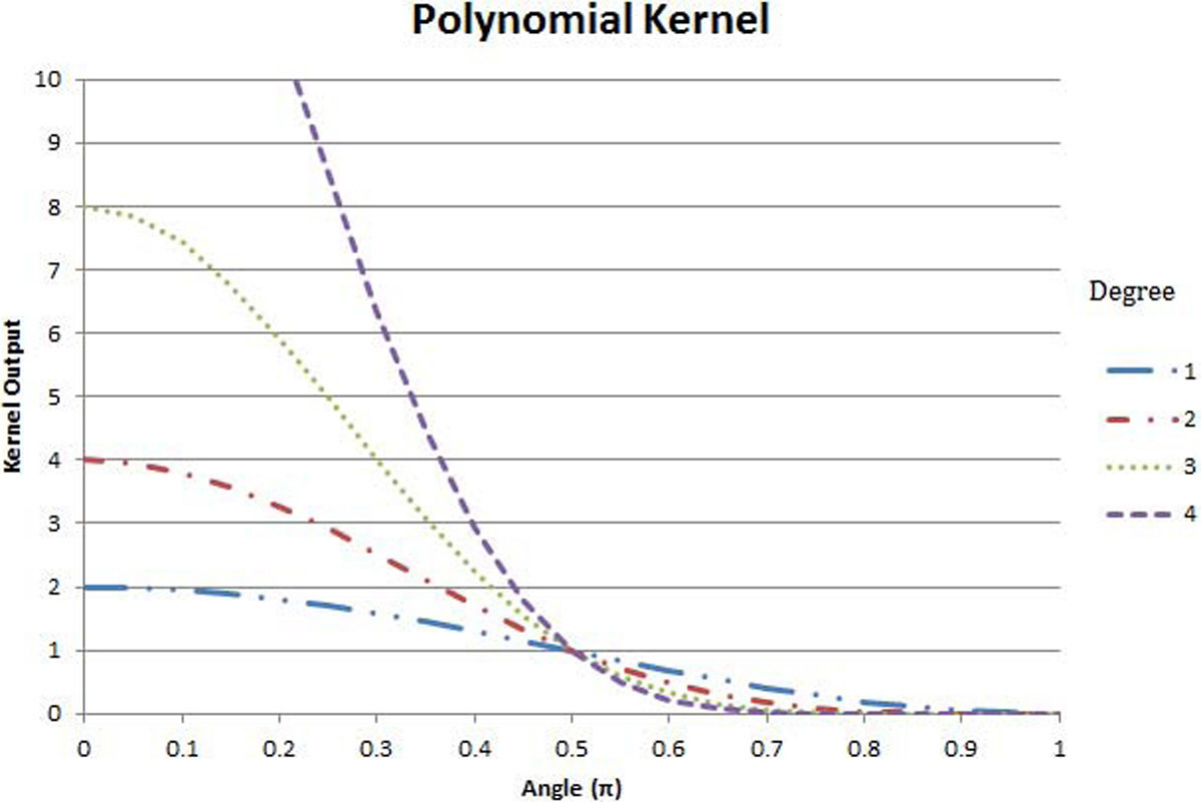
**Polynomial Kernel**. The outputs of the polynomial kernel as functions of the cosine of the angles between vectors. Three functions are depicted in dashed-double-dotted-blue, dashed-single-dotted-red, dotted-green, and dashed-purple curves, corresponding to the cases where the polynomial degree is 1, 2, 3, and 4 respectively.

The dot and polynomial kernels are given by,

(11) $$K\left(\vec{u},\vec{v}\right)=\vec{u}\cdot \vec{v}=∥\vec{u}∥∥\vec{v}∥cos\left(\theta \right),$$

(12) $$\text{and }K\left(\vec{u},\vec{v}\right)={\left(\vec{u}\cdot \vec{v}+1\right)}^{d},$$

respectively, both use the dot product (and therefore the angle between the vectors) to express similarity; however, the input vectors to the polynomial kernel must be normalized (*i.e.*, unit vectors). This restricts the range of the dot product in (12) to ±1, yielding kernel outputs between *0 *and 2^*d*^, where *d *is the degree of the polynomial. The implication of the dot product kernel having a positive and negative range (versus the strictly non-negative polynomial kernel) is that the classification process can learn from the unknown vector's dissimilarity to a known sample, rather than just its similarity. While the dot product kernel will give relatively equal consideration to similar and dissimilar input vectors, the polynomial kernel will give exponentially greater consideration to those cases which are very similar than those that are orthogonal or dissimilar. The value of *d *determines the relative importance given to the more similar cases, with higher values implying a greater importance. Measures of similarity for these two kernels are depicted in Figures 4 and 5.

The Gaussian and Exponential RBF kernels are given by:

(13) $$K\left(\vec{u},\vec{v}\right)=e^{-\frac{∥\vec{u}-\vec{v}{∥}^{2}}{2\sigma^{2}},}$$

(14) $$\text{and }K\left(\vec{u},\vec{v}\right)=e^{-\frac{∥\vec{u}-\vec{v}∥}{2\sigma^{2}},}$$

respectively.

**Figure 6:**
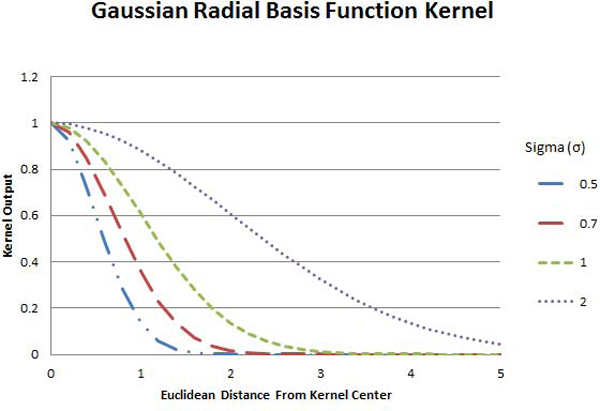
**Gaussian RBF kernel**. The outputs of the Gaussian radial basis function kernel as functions of the Euclidean distance between vectors. Four functions are depicted in dashed-double-dotted-blue, long-dashed-red, dashed-green, and dotted-purple curves, corresponding to the cases where the sigma is 0.5, 0.7, 1.0, and 2.0 respectively.

**Figure 7:**
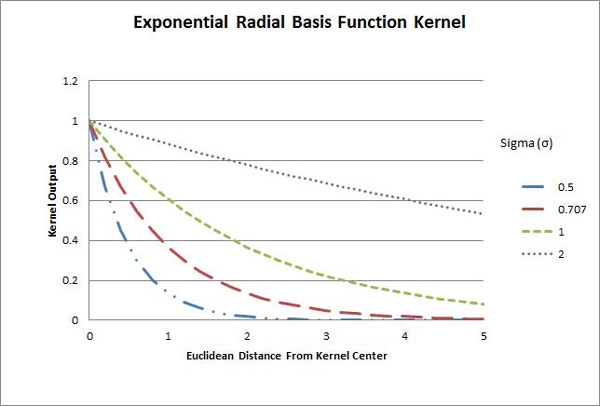
**Exponential RBF kernel**. The outputs of the exponential radial basis function kernel as functions of the Euclidean distance between vectors. Four functions are depicted in dashed-double-dotted-blue, long-dashed-red, dashed-green, and dotted-purple curves, corresponding to the cases where the sigma is 0.5, 0.707, 1.0, and 2.0 respectively.

The Gaussian and Exponential RBF kernels use the Euclidean distance between the two input vectors as a measure of similarity instead of the angle between them (see Figures 6 and 7).

Since ||u - *v*|| is always non-negative, both kernels achieve a maximum output of one when ||u - *v*|| = 0, and approach zero as ||*u *- *v*|| increases. This approach is made faster or slower by smaller or larger values for *σ*, respectively. Figure 6 shows the output of the GRBF kernel as a function of the distance between the input vectors for several different values of *σ*. Figure 7 shows the output of the ERBF kernel.

It is clear from Figures 6 and 7 that the distance at which the kernel output reaches approximately zero varies with σ, and therefore the choice of *σ *for this kernel is essential in properly distinguishing the level of similarity between two input vectors. If the value of *σ *is too small-that is, most pairs of vectors are far enough apart that the kernel output is near zero, the SVM will have too little information to make an accurate classification. If the value of *σ *is too large, so that even very distant pairs of input vectors produce a moderate output, the decision surface will be overly smooth. This may mask smaller distinctive characteristics which exist in the ideal decision surface, and will also increase the effect outliers in the training data have on the classification of an unknown point.

### Using PLS, KPLS, and SVM in clinical research

While the methods covered in this paper offer statistically significant improvements in diagnostic and prognostic biomedical applications, there has been great difficulty in utilizing advances such as these in clinical research. The statistics used to evaluate the performance of these techniques are not readily converted into direct clinical information that may help in patient care or pharmaceutical research. In order to address this, we have devised a framework to combine these techniques with well accepted and understood traditional biostatistics methods, the Cox Proportional Hazard model and the Kaplan-Meier (K-M) Curve. These two techniques each help address the question of how important a particular parameter is to evaluating risk/survival. The following subsections will give a basic overview of how Cox and K-M can be combined with our techniques. For simplicity, such a combination will only be described with PLS, though it could just as easily be done with KPLS or SVM.

#### PLS and Kaplan-Meier curves

Developed in the 1950s, the K-M curve is the gold standard in survival analysis [34]. In a normal survival curve, the number of survivors at a particular moment in time is divided by the total number of patients. These points are plotted against time to give a curve which starts at 1 and slowly curves downward until at some time when it reaches 0. A K-M curve introduces an additional element, the ability to utilize censored data. Censored data is partial data; where a final survival time is unknown but a minimum survival time is known. This can happen when patients die of unrelated causes, patient data is lost, or patients no longer keep contact with the researcher. To handle this censored data, when the partial survival time is reached, the patients are removed from the number of survivors *and *the total number of patients. These removals are marked on a K-M curve with a cross. The best way to utilize a K-M curve is to create different curves for different groups and compare them. For instance, a K-M curve for men and one for women would be far apart if a particular condition was much more fatal in one gender than the other. Using this concept with our techniques, we can use the PLS (or KPLS/SVM) to split a data set of patients into good and poor prognosis categories. This can be done by first splitting training data around some cut-off survival time (survival being the lack of recurrence), such as survival before or after 36-months, and training the system to make predictions on a validation set. K-M curves can then be made for those predicted groups, and if the difference between them is significant, then the system is performing well. A chi-square test is the standard for comparing curves, and a *p*-value derived from that test of below .05 would indicate statistically significant difference between the two prognosis categories predicted by PLS.

#### PLS and Cox Hazard Ratios

Another common survival analysis technique is the Cox Proportional Hazard model [35]. The Cox model is a semi-parametric linear regression model which assumes that the hazard of an observation is proportional to an unknown "baseline" hazard common to all observations. Proportionality to this baseline, it is modeled as an exponential of a linear function of the covariates. From this model, a single Cox Hazard Ratio (CHR) value is derived which represents the "risk" of an event occurring associated with being in a particular group. The larger the CHR, the greater the risk over time of the event occurring for one group than the other. Similar to the K-M curve, the PLS can separate patients into two prognosis categories, and the CHR will be a measure of the effectiveness of that categorization. A large ratio would indicate that the output of this method was a useful prognostic prediction for a patient to have recurrence.

## Results

The goal of the experiments discussed herein were to derive models from the microarray data to classify each sample as belonging to either the class of recurrent or non-recurrent patients. The class of non-recurrent samples are those samples belonging to patients which, after being treated did not recur cancer before the given cut-off period. Patients that did recur cancer before the cut-off period are considered to belong to the recurrent class. Two separate experiments were performed with cut-off periods of 36 and 60 months respectively.

As mentioned in the Methods section, the data were pre-processed using CFR, followed by FFS, and finally classification model building and evaluation.

### Coarse Feature Reduction

For the 36 month classification experiment, CFR was used to reduce the original number of features (probes) from 22,282 to 2,675 using a hard cut-off *t*-test *p*-value of 0.05. Then, this probe count was further reduced to 594 using a coefficient of variation cut-off of 0.632. In like manner, using CFR for the 60 month classification experiment, the number of probes was reduced from 22,282 to 829 using the same hard cut-off *t*-test *p*-value of 0.05. This number was then further reduced to 212 using coefficient of variation cut-off of 0.641. After reducing the initial feature set using the CFR technique, the process of FFS and classification was performed.

### Fine Feature Selection/Classification

#### Fine Feature Selection using Partial Least Squares

In this section, we use the AUC value as the fitness metric to evaluate the relative worth of the classification model. Higher AUC values are indicative of better classifiers, with an AUC value of 1.0 indicating a perfect classifier, which is arguably impossible for any non-trivial classification task.

The FFS process utilizes the weight vector of the first latent variable generated by the Linear PLS (L-PLS) algorithm to ascertain feature importance. The most important features (those with the largest corresponding weight vector components) are ranked highest and features with lower corresponding components are discarded. This step, called Fine Feature Selection, provides a ranking of importance, which means the magnitude of each feature's respective component is directly correlated with its predictive power in the model.

The FFS process builds this "importance metric" by iterating the analysis of the weight vectors of randomly assigned training folds 10,000 times employing three sensitivity settings, where these three sensitivities score the top 20, 30, and 150 most influential performers for each of their respective 10,000 runs, based on each feature's weight in the weight vector of L-PLS. For example, if 'Age' has the largest component and 'Sex' has the second largest in the top 30 sensitivity setting, the score for 'Age' would be 30 and that for 'Sex' would be 29. For each run time, the data is split randomly into training and validation folds. These data are normalized then analyzed using Linear PLS and the weight vector is extracted, sorted, and 'winning' features have their scores updated by position.

In each of the three settings, a number, *p*, of features are retained based on theirs aggregated score over 10000 runs. The number to retain, *p*, is user-specified. In FFS, we tried several *p *values, with increments of 50 features, beginning at 50 and ending at 550. By gradually increasing the size of feature retention, one can empirically optimize the number of features for classification/prediction. Lastly, a 'global (most important) feature set (*S*_*FFS*_)' is created, which is the union of the retained feature sets from all three settings. These *S*_*FFS *_features are the final product of the FFS process and the only ones included in the construction of the refined input data matrix, *X*_*FFS*_. In summary, *S*_*FFS *_is given by:

(15) $$S_{FFS}^{p}=S_{20}^{p}⋃S_{30}^{p}⋃S_{150}^{p}$$

where $S_{FFS}^{p}$ = the union set of all three top performing feature subsets, $S_{l}^{p}$ = each setting's top performers, *l *= 20, 30, 150, and *p *= the number of features retained in each setting. Note that the number of features in $S_{FFS}^{p}$ may not be exactly 3*p *or *p*.

In our study, we have selected 361 and 102 features using this FFS process for the 36- and 60-month experiment respectively, from the 594 and 212 features that were selected by CFR.

#### Comparisons using PLS classification

As noted, we compared four separate models' performances based on different data: L-PLS and K-PLS Polynomial Kernel (KPLS-Poly) based on the Coarse Feature Reduced (CFR) data, and on the Fine Feature Selected (FFS) data respectively (the FFS-data is actually processed by both CFR and FFS).

• We sought out to determine which model produced the most accurate prediction of recurrence.

• We also sought to determine whether the data was linear or non-linear, which was determined by which class of model yielded better results: L-PLS or K-PLS with non-linear kernels.

• Finally, we sought to determine the effectiveness of our *PLS weight vector-based *Fine Feature Selection method. This was determined by the comparison between the validation AUC values for the same models on the CFR-data and the FFS-data. If the results on the FFS-data are better than the CFR-data, then FFS is effective.

What we found was that both the 36-month and 60-month data sets were inherently linear in nature, meaning the L-PLS gave better AUC values on validation folds. These results can be seen in Table 1. This is a particularly surprising find, considering most real world phenomena are non-linear by nature. Yet, this was verified by our K-PLS Evolutionary Programming optimization technique which selected polynomial kernel parameter of degree 1 as the best performer (K-PLS with polynomial kernel whose degree equals 1 is equivalent to L-PLS.) The validation AUC values, as we will show, were near equivalent for L-PLS and the KPLS-Poly of degree 1. As a second verification of our results, a Support Vector Machine (Lib-SVM) [36] analysis on the same data supported our findings by producing the same validation AUC values with the polynomial kernel. The LibSVM analysis also supported our pre-analysis which showed extremely poor performance of the Gaussian/Exponential RBF kernels on these data sets. Due to this inadequate performance, we did not continue our study with the RBF kernels.

**Table 1:** Model Comparison The comparison of optimal performance values and number of latent variables for three independent models on the 36- and 60-month data.

(CFR-data) Model	Top Validation AUC Value (36 mo/60 mo)	Number of Latent Variables (30 mo/60 mo)
L-PLS	.791/.831	3/2

KPLS-Poly (Degree = 1)	.784/.830	3/1

SVM	.78/-	-

The best performance was seen with the L-PLS, out-competing the non-linear SVM and KPLS techniques in AUC performance. The number of latent variables required for the PLS-based techniques was no more than three for both data sets.

**Figure 8:**
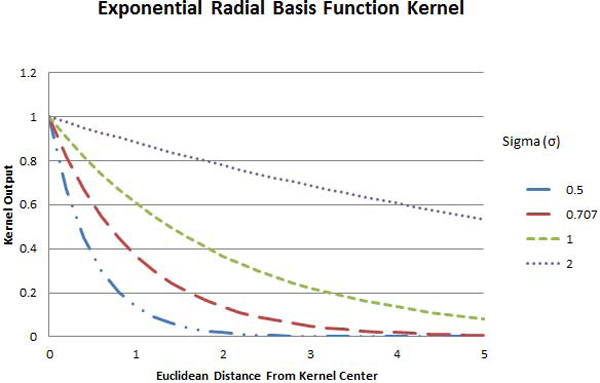
**36-mo. Training AUC Values vs. Latent Variables**. 36-mo. Training AUC Values vs. Latent Variables

**Figure 9:**
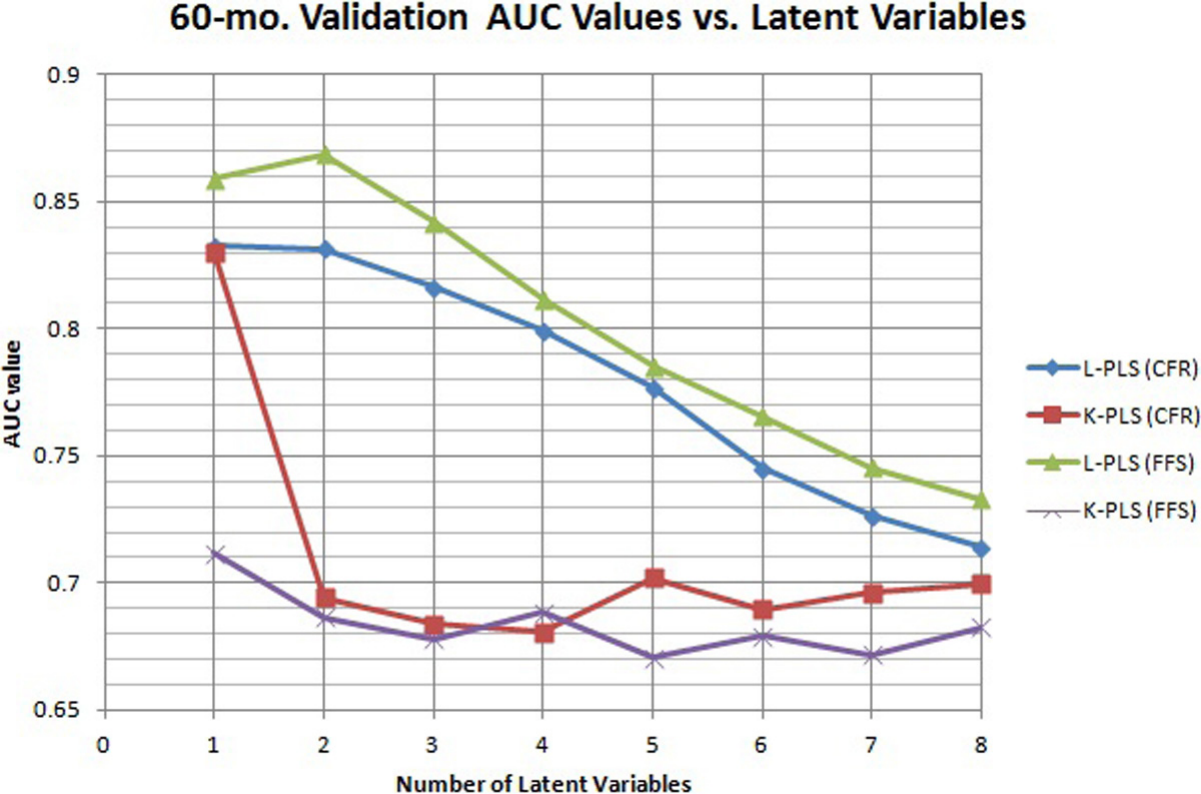
**60-mo. Training AUC Values vs. Latent Variables**. 60-mo. Training AUC Values vs. Latent Variables

In addition to these findings, the number of latent variables required to reach optimal performance, by L-PLS and KPLS-Poly when they are applied to the FFS processed data was roughly the same (see Figures 8 and 9 for 36 months and 60 months respectively). In the case of 36 months, the best number of latent variables is 3 (Figure 8) for both L-PLS and KPLS-Poly models. For the 60-month data set (Figure 9), the KPLS-Poly had a slightly lower required number, 1, for latent variables than the PLS model, which requires 2. As is also shown in Table 1, the KPLS-Poly reached the maximum performance at 1 latent variable whereas the L-PLS reached maximum at 2 latent variables for the 60-month experiment. This means that the KPLS-Poly analysis did not require as much smoothing of the data to reach its optimal validation AUC value. This is also indicative that the best system generalization was seen between 1 and 3 latent variables, which is typical to most data analyses using these techniques (most are less than 5).

The analysis of the efficacy of our *PLS weight vector-based *FFS technique in reducing noisy features shows that is effective only for the L-PLS method. The results can be seen in Table 2. In both the 36-and 60-month datasets, top performance was only improved in terms of AUC value for the L-PLS. It is to our belief that this is due to the fact that we base our FFS of the features on their linear combination of the contributions that they have to the time of recurrence. We believe that the use of a KPLS-based method embedded in the FFS process would capture those features which show non-linear contributions to the response variable. The KPLS-Poly model was, in both cases, impacted by the removal of some features which must have had a critical role in its classification model. This was seen more severely in the 60-month data set, may be due to the fact that it, from the beginning, had half the amount of features than the 36-month after CFR.

**Table 2:** CFR and FFS Comparison The comparison of model performance on data from the Fine Feature Selection process and the Coarse Feature Reduction.

Model	Top Validation AUC Value CFR-data (36 mo/60 mo)	Top Validation AUC Value FFS-data (36 mo/60 mo)
L-PLS	.791/.831	.794/.869

KPLS-Poly (Degree = 1)	.784/. 830	.780/.711

The FFS process enhanced performance for only the L-PLS while the KPLS-Poly suffered in both the 36- and 60-month data.

#### SVM Verification of K-PLS polynomial results

The 36 month KPLS-Poly AUC result of 0.784 was not expected when compared with the L-PLS result AUC result of 0.791 because these classification problems are generally non-linear. We therefore validated this result with an independent analysis using SVM using several kernels with the exact same data set and cross-validation process. Specifically, the data was normalized and formatted for use with LibSVM [36], a widely-used SVM implementation. A grid search was implemented to find good parameters for each of the built-in kernels, Gaussian RBF, Sigmoid, and polynomial. A linear SVM was not considered as it would not be a good comparison to K-PLS. With the grid search including four parameters: gamma, coefficient, degree, and C (regularization parameter), the polynomial kernel was found to be the best performer. Using 1-hold-out cross-validation, the best results found by this method was ~0.78 (which agrees with the K-PLS polynomial result to within 0.51%), though most parameter configurations usually gave an output of .63-.73. No stochastic optimizer was used, so it may be possible for slightly higher performance (and slightly better agreement) with a exhaustive EP parameter search. Other results in Table 2 above were not verified because of the exhaustive analysis performed for this 36 month K-PLS polynomial result.

### Kaplan-Meier and Cox

**Figure 10:**
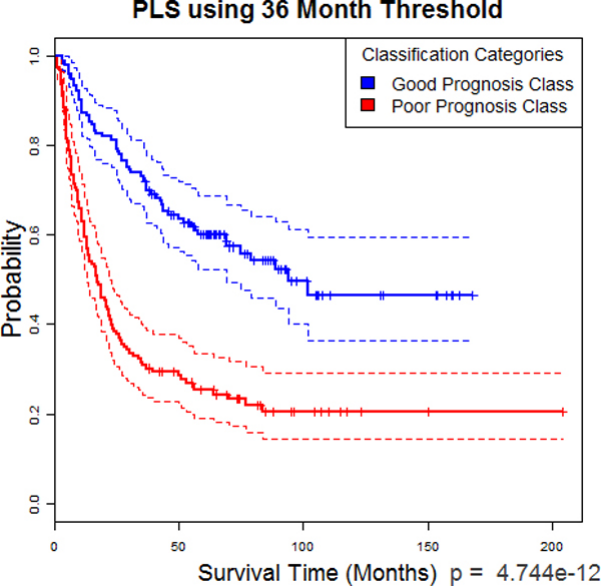
**PLS at 36 Month Threshold**. Kaplan-Meier curve of PLS predicted groups using 36-month threshold

**Figure 11:**
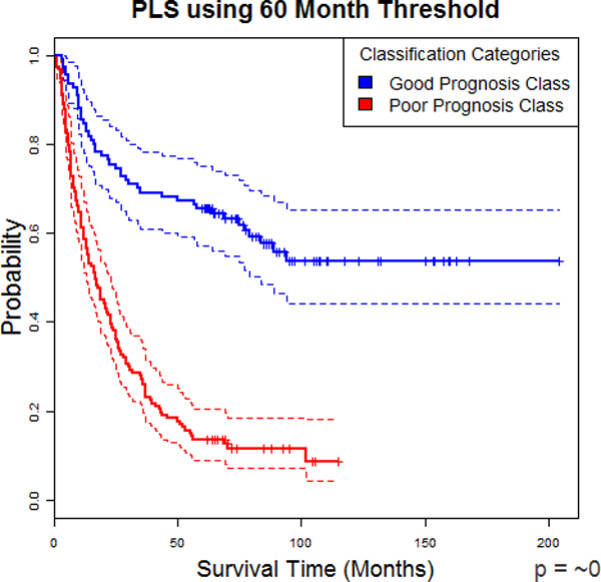
**PLS at 60 Month Threshold**. Kaplan-Meier curve of PLS predicted groups using 60-month threshold

K-M curves for both PLS using 36 and 60 months can be seen in Figures 10 and 11 respectively. Using 36 months as the cut-off for training, the resulting K-M curves for the two categories have a very significant difference of *p *= 4.744e-12. For 60 months, the *p*-value was so low as to only give ~0 as a result of calculations. A higher precision computation tool may be capable of a more specific result. However, these results make it very clear that PLS is easily able to separate patients into groups of recurrence sooner and later. The CHR between the two categories using the 36-month cut-off for training was 2.846341 (2.088547 and 3.879089 for 95% confidence). For 60-months, it was 3.996732(2.828351 and 5.647768 for 95% confidence). These numbers show a significantly increased risk of recurrence over time with being in the poor prognosis group versus the good prognosis group. These two statistics, the K-M curve derived *p*-values and CHR, are values which can be directly understood by clinicians without further training. In other words, with this framework, any new patient's data could be sent to us by any doctor who reads this article, given a categorization by the system as it is currently setup, and then the doctor can take that knowledge and make decisions on how frequent to make checkups and other treatment decisions.

## Conclusions

Our microarray analysis and information extraction method comprised three basic components drawing from Statistical Learning Theory: 1.) Coarse Feature Reduction, 2.) Fine Feature Selection and 3.) Classification.

In Coarse Feature Reduction, the original 22,282 probes were reduced to 594 for the 3 year cut-off (97.5% reduction) and to 212 for the 5 year cut-off (99.04% reduction) using basic t-test and variance pruning techniques. The Fine Feature Selection was able to further reduce the number of features to 361 for the 60-month and 102 for the 36-month data sets (a further reduction of 39.2% and 51.9%). The FFS process has been demonstrated to reduce the noise in the data by filtering out noisy features from the data set produced by the CFR process. By implementing the FFS process in our analysis, we were able to enhance the performance of our classifier.

After utilizing the FFS process, classification comparison is made for the refined data. The optimal classifying performance of L-PLS was observed at 3 latent variables and 2 latent variables for the 36- and 60-month experiments, respectively. Similar results were obtained, a reduction to 3 and 1 latent variables, when using L-PLS on data refined only by CFR. The Area Under the Curve (AUC) measure of performance varied from 0.791 to 0.869, depending upon the particular L-PLS or K-PLS and SVM model used (see Tables 1 and 2). PLS results for the 36-month cut-off were independently verified using Support Vector Machines. In summary, it is important to note that by using the SLT techniques, over 22,000 probes were eventually reduced to 3 and 2 latent variables (for the 36- and 60-month cut-off periods, respectively) while still maintaining AUC values in the range of 0.79 to 0.86.

This research also provided a secondary and clinically important result, which is that the improved SLT methods/paradigms can be integrated into the widely accepted and well understood traditional bio-statistical Cox Proportional Hazard model and the K-M methods. For example, using the SLT paradigms as pre-processors for K-M, the resultant probability vs. survival time categories have a very significant difference (*p *= 4.74*e*-12) for the 36-month cut-off and a *p *~0 for the 60-month cut-off. (Figures 10 and 11, respectively). These results, therefore, make it clear that PLS easily and accurately separates patients into groups of sooner and later recurrence. Furthermore, the CHR between the two categories for the 36-month cut-off was 2.85 (2.09 to 3.88 for 95% confidence). For the 60-month cut-off the ratio was 3.99 (2.83 to 5.65 for 95% confidence). (Figures 10 and 11, respectively). These results show a significant increased risk of recurrence over time when classified as being a member of the poor group vs. the good group. Consequently, these two results (K-M derived *p*-values and the CHR), which are directly understood by practicing clinicians without additional training and were pre-processed using the PLS and KPLS algorithms, was made possible by the SLT pre-processing we applied in this study.

## Competing interests

The authors declare that they have no competing interests.

## Authors' contributions

WHL directed the overall effort and WHL and XQ developed the new and refined existing (as appropriate) statistical learning theory and complex adaptive systems approaches employed in this paper. WSF, DEM, CTP and JFPR were involved in the experimental design used to ascertain the efficacy of these SLT algorithms in assessing the treatment of non-small cell lung cancer. WHL, WSF, DEM, XQ, CTP and JFPR were involved in the results analysis and provided many useful scientific insights. YD coordinated and directed the whole project. YD, JYY and JAB provided the data sets and provided clinical insight/analysis for these data sets. All co-authors participated in the final analysis and reviews of the results of these experiments and agreed on the content of the paper.
